# Barriers to hand hygiene practices and improvement expectations among awake ICU patients: a qualitative study

**DOI:** 10.3389/fpubh.2026.1814268

**Published:** 2026-06-02

**Authors:** Yinping Yi, Tiantian Wu, Chan Jing, Xiaoning Du, Yu Shan, Qiuhuan Jiang, Linlin Hou, Lin Tao

**Affiliations:** 1 Cardiac Intensive Care Unit, Henan Provincial Key Medicine Laboratory of Nursing, Henan Provincial People’s Hospital, Zhengzhou University People’s Hospital, Zhengzhou, Henan, China; 2 Infectious Diseases Intensive Care Unit, Henan Provincial Key Medicine Laboratory of Nursing, Henan Provincial People’s Hospital, Zhengzhou University People’s Hospital, Zhengzhou, Henan, China; 3 Nursing Department, Henan Provincial Key Medicine Laboratory of Nursing, Henan Provincial People’s Hospital, Zhengzhou University People’s Hospital, Zhengzhou, Henan, China

**Keywords:** behavioral determinants, COM-B model, hand hygiene, infection prevention, intensive care unit, patient engagement

## Abstract

**Background:**

Awake patients in intensive care units (ICUs) retain partial self-care ability and may contribute to pathogen transmission through contaminated hands. However, patient hand hygiene is insufficiently emphasized, and adherence remains low. A deeper understanding of patients’ behavioral challenges and support needs remains lacking.

**Objectives:**

This qualitative study aimed to explore the barriers to hand hygiene practices among awake ICU patients and identify their improvement expectations.

**Methods:**

A qualitative descriptive study was conducted in a tertiary hospital in Henan Province, China. Fifteen awake ICU patients participated in semi-structured interviews between December 2024 and April 2025. Data were analyzed using deductive content analysis informed by the Capability–Opportunity–Motivation Behavior (COM-B) framework.

**Results:**

Fifteen awake ICU patients were interviewed. Two overarching themes emerged with 17 subcategories: (1) barriers to hand hygiene practices, including incomplete knowledge, physical burden, cognitive fluctuations, limited resource accessibility, environmental constraints, lack of social support, insufficient health education, low perceived infection risk, emotional burden, diminished self-efficacy, unclear role perception, and insufficient emphasis from healthcare providers; and (2) improvement expectations for hand hygiene practices, including clear behavioral boundaries, ability-adapted health education, sustainable behavioral prompts, improved accessibility, role clarification with positive feedback, and respectful communication and care practices.

**Conclusion:**

Awake ICU patients experience multiple barriers to hand hygiene related to physical condition, environmental support, and motivation to engage during critical illness. Promoting patient participation in hand hygiene may therefore require not only education, but also individualized support, accessible resources, and respectful communication. These findings may inform future efforts to improve patient hand hygiene in ICU settings.

## 1 Introduction

Healthcare-associated infections (HAIs) remain a major global threat to patient safety, particularly in intensive care units (ICUs), where infection incidence is substantially higher than in general wards (1, 2). Preventing HAIs in ICU settings therefore remains a central priority in infection control and public health. Growing evidence suggests that hospitalized patients’ hands may harbor pathogenic microorganisms and contribute to environmental contamination and transmission (3–5). Several studies have reported reductions in healthcare-associated infection rates following improvements in patient hand hygiene (6, 7), and current guidance from the U.S. Centers for Disease Control and Prevention (CDC) recommends hand hygiene as a fundamental infection prevention practice for both healthcare personnel and patients in healthcare settings (8). Recent acute-care research further indicates that patients with severe functional dependence are more likely to harbor multidrug-resistant organisms (MDROs) on their hands (9). Nevertheless, patient hand hygiene adherence remains suboptimal. A cross-sectional survey involving 310 hospitalized patients and accompanying caregivers in China reported suboptimal patient hand hygiene practices, with 62.3% of respondents washing their hands fewer than five times daily and poorer hand hygiene practice among ICU patients than medical ward patients (10). However, patients’ understanding of specific hand hygiene moments in ICU contexts remains insufficiently explored. Taken together, these findings underscore the need to better understand the factors influencing patient hand hygiene practices in ICU settings.

Despite increasing attention to patient hand hygiene, existing studies have primarily quantified adherence levels or implemented education- and reminder-based interventions, largely within general ward populations (10–13). These studies suggest that patients’ knowledge of key hand hygiene moments, availability of bedside resources, and reminders from healthcare workers may influence patient hand hygiene practices. However, they provide limited insight into the behavioral determinants underlying patient hand hygiene practices in ICU settings. In ICU environments characterized by critical illness, invasive devices, restricted mobility, and continuously evolving treatment demands, awake patients often retain partial self-care capacity and continue to interact with healthcare providers and surrounding surfaces. Yet, how they perceive hand hygiene and engage in infection prevention under such constraints remains insufficiently understood. The Capability–Opportunity–Motivation Behavior (COM-B) model provides a structured framework for examining how individual capability, environmental opportunity, and motivational processes interact to influence behavior (14). Guided by this model, the present qualitative study aimed to explore the barriers to hand hygiene practices and improvement expectations among awake ICU patients in order to inform the development of context-sensitive strategies for promoting patient hand hygiene in ICU settings.

## 2 Methods

### 2.1 Design

This study adopted a qualitative descriptive design to explore the barriers to hand hygiene practices and improvement expectations among awake ICU patients. Qualitative descriptive methodology is particularly suitable for obtaining rich descriptions of participants’ experiences in clinical contexts. The study was reported in accordance with the Consolidated Criteria for Reporting Qualitative Research (COREQ) guidelines (15), to enhance methodological transparency and reporting rigor (see Supplementary file 1).

### 2.2 Theoretical framework

This study was guided by the Capability–Opportunity–Motivation Behavior (COM-B) model, developed by Michie et al. (14). The COM-B model posits that behavior is shaped by the interaction of capability, opportunity, and motivation (16). Capability includes physical capability (e.g., bodily function and physical skills) and psychological capability (e.g., knowledge, understanding, memory, and decision-making). Opportunity includes physical opportunity (e.g., environmental conditions, available resources, and time) and social opportunity (e.g., interpersonal influences, social norms, and social support). Motivation includes reflective motivation (e.g., beliefs, intentions, evaluation, and planning) and automatic motivation (e.g., emotions, impulses, habits, and affective responses). In this study, the COM-B framework guided the development of the interview guide, informed the initial coding framework for deductive content analysis, and supported the interpretation of barriers and improvement expectations related to hand hygiene among awake ICU patients.

### 2.3 Setting and participants

This study was conducted in the adult intensive care units of a tertiary hospital in Henan Province, China, between December 2024 and April 2025. The hospital has multiple adult ICU units with different specialty orientations, where patients commonly experience restricted mobility, invasive treatments, and varying levels of dependence on nursing care. In these units, bedside caregiving by family members is generally restricted, and family visits are typically limited to scheduled daily visiting periods. Purposive sampling was used to recruit awake ICU patients from adult ICU units. The inclusion criteria were: (1) age ≥18 years; (2) ICU stay ≥24 h; (3) awake and clinically stable, defined as a Glasgow Coma Scale (GCS) score ≥14 and a Richmond Agitation–Sedation Scale (RASS) score of 0; (4) able to communicate effectively and describe hand hygiene-related experiences during ICU hospitalization; and (5) provision of written informed consent. The exclusion criteria were: (1) severe cognitive impairment, psychiatric disorders, or delirium that hindered communication; (2) unstable vital signs that contraindicated participation in interviews; and (3) severe hearing or speech impairments that affected communication. Sampling continued until data saturation was achieved. A total of 17 eligible patients were approached; two declined participation because of fatigue. Fifteen patients were enrolled in the study.

### 2.4 Data collection

Data were collected through semi-structured, face-to-face interviews. The interview guide was developed based on a literature review and discussions within the research team and was refined following feedback from two pilot interviews with awake ICU patients. The guide was developed in alignment with the three core components of the COM-B model—Capability, Opportunity, and Motivation. Open-ended questions were used to explore patients’ knowledge, perceived ability, environmental influences, social support, beliefs, emotional responses, and attitudes toward hand hygiene during ICU hospitalization. During the interviews, additional probing questions were used as needed to further explore participants’ responses and clarify issues related to the COM-B domains. The main interview questions included: (1) What is your understanding of hand hygiene? (2) How do you think hand hygiene in the ICU differs from your usual hand hygiene habits in daily life? (3) Are there any situations in the ICU where you feel it is particularly important to clean your hands? (4) What challenges, if any, have you encountered when trying to clean your hands in the ICU? (5) Can you recall a time when you wanted to clean your hands but could not? What was happening at that time? (6) How do you feel about the support you receive from healthcare workers or family members in helping you with hand hygiene? (7) What changes or improvements would make it easier for you to clean your hands in the ICU? (8) Is there anything about hand hygiene in the ICU that you would like to share that has not been mentioned yet? All interviews were conducted by the first author (YY), a female researcher with ICU clinical experience and formal training in qualitative research. There was no prior relationship between the interviewer and the participants. Before each interview, participants were informed of the study objectives, procedures, and ethical considerations and were assured that confidentiality would be strictly maintained, with personal identifiers replaced by codes. Interviews were conducted at the patients’ bedside during relatively quiet periods in the ICU to minimize interruptions. Each interview lasted approximately 30–40 min and was audio-recorded with participants’ permission. During the interviews, the researcher maintained a neutral stance, avoided leading questions, and encouraged participants to freely express their hand hygiene experiences throughout their ICU stay, rather than only their status at the time of interview. Paraphrasing and clarification were used where needed to elicit rich and detailed accounts. Field notes were taken to document nonverbal cues and relevant contextual observations.

### 2.5 Data analysis

Data were analyzed using deductive content analysis informed by the COM-B model, supported by NVivo 12 software. Audio recordings were transcribed verbatim within 24 h and cross-checked against field notes for accuracy. The COM-B model served as an *a priori* analytical framework, and three initial coding domains—Capability, Opportunity, and Motivation—were established before formal coding. Two researchers (YY and TW), both trained in qualitative research, independently read the transcripts multiple times to achieve immersion in the data. Meaning units relevant to patients’ hand hygiene experiences were identified, and initial semantic coding was conducted at the semantic level to preserve participants’ original meanings. The resulting codes were then deductively organized within the predefined COM-B domains. Within each domain, codes with similar meanings were compared, refined, and grouped into subcategories and categories through iterative analysis. Discrepancies in coding and categorization were resolved through discussion, and unresolved disagreements were reviewed with a third researcher (XD). The coding framework was continuously refined throughout the analytic process. Data saturation was considered achieved when no new codes or categories emerged from additional interviews. Representative quotations were selected to illustrate each category and theme.

### 2.6 Ethics statement

This study was approved by the Ethics Committee of Henan Provincial People’s Hospital (Approval No. 2022-123). Written informed consent was obtained from all participants prior to data collection.

### 2.7 Rigor and reflexivity

To enhance rigor, interviews were conducted by the first author (YY), an ICU nurse with qualitative research training. Familiarity with the ICU setting facilitated rapport-building and contextual understanding during interviews. Reflexive discussions within the research team were conducted throughout data collection and analysis to minimize the influence of prior assumptions on data interpretation. Field notes documenting contextual observations and researchers’ reflections informed the interpretation of participants’ experiences. A coding framework and analytic memos were maintained throughout the analytic process. Due to participants’ clinical vulnerability and limited availability during ICU hospitalization, transcripts were not returned for member checking. Instead, credibility was enhanced through team discussions, analytic documentation, and the use of representative quotations to strengthen the trustworthiness of the findings.

## 3 Results

This study included 15 awake ICU patients aged 26–79 years. Participant characteristics are summarized in Table 1. Guided by the COM-B model, the analysis generated two overarching themes: barriers to hand hygiene practices and improvement expectations for hand hygiene practices. These themes comprised six categories, 17 subcategories, and 44 codes. A summary of the analytic structure within the COM-B framework is presented in Table 2.

**Table 1:** General information of participants (*n* = 15).

No.	Gender	Age (years)	Occupation	Education level	Marital status	ICU stay duration (days)
N1	Male	67	Retired	Bachelor’s degree	Married	7
N2	Female	48	Civil servant	Vocational/technical school	Married	10
N3	Male	71	Retired	Primary school	Married	7
N4	Female	39	Self-employed	Master’s degree	Married	8
N5	Female	77	Agriculture	Primary school	Widowed	10
N6	Male	51	Healthcare professional	Master’s degree	Married	6
N7	Female	65	Retired	Junior high school	Married	4
N8	Male	26	Student	Master’s degree	Unmarried	6
N9	Female	28	Teacher	Bachelor’s degree	Unmarried	12
N10	Female	67	Retired	Vocational/technical school	Married	7
N11	Male	79	Agriculture	Primary school	Married	5
N12	Male	35	Self-employed	Bachelor’s degree	Married	4
N13	Female	68	Agriculture	Primary school	Married	6
N14	Male	57	Retired	Junior high school	Married	6
N15	Male	48	Self-employed	High school	Divorced	3

**Table 2:** Analytic structure of barriers and improvement expectations related to patient hand hygiene based on the COM-B model.

Overarching theme	COM-B domain	Subcategories
Barriers to hand hygiene practices	Capability	Incomplete knowledge framework regarding hand hygiene; Physical burden and reduced functional capacity; Fluctuations in cognitive capacity
	Opportunity	Limited accessibility to hand hygiene resources; Environmental constraints in the ICU; Lack of social support; Insufficient health education implementation
	Motivation	Insufficient perception of infection risk; Perceived insufficient emphasis on patient hand hygiene by healthcare providers; Emotional burden and psychological exhaustion; Lack of self-efficacy and ambiguity in role perception
Improvement expectations for hand hygiene practices	Capability	Clear and safe boundaries for initiating behaviors; Health education tailored to current abilities
	Opportunity	Sustainable and easy-to-trigger behavioral prompts; Improving accessibility through environmental and procedural optimization
	Motivation	Enhancing motivation through role clarification and positive feedback; Respectful communication and care practices

### 3.1 Barriers to hand hygiene practices

#### 3.1.1 Capability-related barriers to hand hygiene practices

##### 3.1.1.1 Incomplete knowledge framework regarding hand hygiene

Some patients conceptualized “hand hygiene” primarily as general hand cleanliness rather than as context-specific practices linked to distinct clinical moments. Their understanding lacked clarity regarding appropriate indications and the selection of hand-cleaning methods, which contributed to uncertainty in complex care contexts and was reflected in delayed, inconsistent, or omitted hand hygiene practices. Participants reported unfamiliarity with the concept of hand hygiene prior to ICU admission, difficulty identifying appropriate moments during hospitalization, and uncertainty regarding the correct use of available hand hygiene products.

*“Before coming to the ICU, I had never heard of ‘hand hygiene.’ I thought it just meant washing hands with water, but I cannot even get up to wash my hands.” (N2).*

*“I know the seven-step hand washing method, but as an ICU patient, I’m not fully clear on when I should perform hand hygiene.” (N6).*

*“Compared with before I got sick, my hygiene is much worse now, but I do not really know how to improve it.” (N1).*

*“The main times I can think of are before eating and after using the toilet.” (N4).*

*“Sometimes I use wet wipes, sometimes I use disinfectant, but I’m not sure what the correct procedure is.” (N11).*

##### 3.1.1.2 Physical burden and reduced functional capacity

Most awake ICU patients were in the acute phase of illness or early recovery and reported varying degrees of physical burden, including reduced upper limb strength, restricted joint mobility, and pain. These physical limitations affected patients’ ability to perform hand hygiene independently. Participants described difficulties completing basic hand hygiene actions, such as pressing a hand sanitizer dispenser or rubbing their hands adequately, particularly when functional capacity was compromised by illness or treatment-related factors. In this context, some patients reported refraining from attempting hand hygiene due to perceived physical difficulty or concern about exacerbating discomfort or risk.

*“My arms feel weak. Even lifting them takes a lot of effort, let alone cleaning my hands properly.” (N4).*

*“There are several tubes attached to me. I’m afraid even moving slightly might pull something out, making it hard to move my hands.” (N1).*

##### 3.1.1.3 Fluctuations in cognitive capacity

Patients reported fluctuations in cognitive capacity during ICU hospitalization. These fluctuations were commonly described as reduced attention, delayed responses, and difficulties with information processing. Participants described challenges in recognizing appropriate moments for hand hygiene and maintaining continuity of hand hygiene behaviors. Even after receiving hand hygiene education, some patients reported difficulty consistently performing recommended practices due to limited memory retention or impaired comprehension.

*“Sometimes my mind is not very clear, and I react slowly. I just cannot remember that I need to clean my hands.” (N10).*

*“I do not sleep well and feel very tense… I’m kind of foggy, and hand hygiene just slips my mind.” (N7).*

*“The nurses explained it, but I could not really remember it.” (N11).*

#### 3.1.2 Opportunity-related barriers to hand hygiene practices

##### 3.1.2.1 Limited accessibility to hand hygiene resources

Although ICU settings are generally equipped with essential hand hygiene supplies, participants reported that the placement of these items frequently changed during hospitalization. For patients with limited mobility or impaired vision, locating or accessing hand hygiene resources in a timely manner was often challenging. Participants described difficulties initiating hand hygiene behaviors when supplies were out of reach, visually obstructed, or inconsistently positioned around the bedside.

*“The disinfectant or wet wipes are always out of my reach.” (*N6*).*

*“The items beside the bed are often moved, and I cannot see them. I do not know where the wet wipes are when I want to use them.” (*N11*).*

##### 3.1.2.2 Environmental constraints in the ICU

Participants described the ICU as a confined and equipment-dense environment, where patients were frequently connected to multiple treatment or monitoring devices, and some were subject to protective restraints. In this context, upper limb movement was often constrained by environmental factors and safety-related monitoring rather than patients’ physical inability. Patients described hesitancy to initiate hand hygiene independently due to concerns about dislodging tubes, triggering alarms, or violating safety precautions. These environmental constraints limited patients’ perceived ability to perform hand hygiene within the ICU setting.

*“My hands are tied (the patient used restraints), and I cannot clean them even if I want to.” (N3).*

*“The space is too cramped, and even turning over is difficult, let alone washing my hands.” (N5).*

*“I was about to sit up, but the machines around me started alarming. I was worried that I might have done something wrong, so I did not dare to move on my own.” (N10).*

##### 3.1.2.3 Lack of social support

The high-intensity care routines in the ICU make it difficult for healthcare staff to consistently provide reminders or assistance. Additionally, family support is limited due to visitor restrictions, which reduces the external support available to patients. In this context, patients often avoid seeking help to prevent burdening the medical staff, and hand hygiene practices are more likely to be neglected.

*“No one reminds me, so I do not know when I should clean my hands.” (N11).*

*“The nurses are very busy, and I do not want to bother them with something like hand hygiene.” (N5).*

*“Family cannot be here, and even though I know I should wash my hands before eating or after using the toilet, I do not have anyone to help me.” (N8).*

##### 3.1.2.4 Insufficient health education implementation

Participants reported limited exposure to hand hygiene–related information within routine health education during ICU hospitalization. Patients described that educational materials provided to them rarely included information specific to hand hygiene, and reminders from healthcare providers were often brief and nonspecific. Participants expressed difficulty understanding appropriate timing and methods for hand hygiene in the absence of detailed or situation-specific guidance.

*“The nurses show me some health education materials, but I’ve never seen anything about hand hygiene. They rarely remind me about it.” (N4).*

*“Sometimes the nurses remind me to clean my hands, but what I really need is for them to explain when and how to do it, not just give me a simple instruction.” (N1).*

#### 3.1.3 Motivation-related barriers to hand hygiene practices

##### 3.1.3.1 Insufficient perception of infection risk

Participants expressed limited perception of infection risk associated with hand contamination in the ICU setting. Some patients underestimated their personal susceptibility to infection and questioned the relevance of patient-performed hand hygiene to their own health outcomes. In this context, hand hygiene was not consistently perceived as a necessary self-protective behavior, particularly when survival or critical treatment outcomes were prioritized over preventive practices.

*“The ICU should be clean; I feel like the medical staff has already taken care of it, so whether or not I do hand hygiene does not matter much.” (N5).*

*“Right now, the most important thing is to stay alive. What difference does it make if my hands are clean?” (N3).*

*“I know hand hygiene is related to infection, but it does not seem to have much to do with my recovery.” (N6).*

##### 3.1.3.2 Perceived insufficient emphasis on hand hygiene by healthcare providers

Participants perceived limited emphasis on patient hand hygiene from healthcare providers during ICU hospitalization. When clinical interactions focused primarily on disease management and monitoring, participants described reduced motivation to initiate or maintain hand hygiene and reluctance to request assistance, particularly when healthcare providers were perceived to be busy or when hand hygiene was not explicitly prioritized in care interactions.

*“They’re busy doing* var*ious treatments, and hand hygiene does not seem to be given much importance.” (N8).*

*“The healthcare providers never really emphasized it; it seems like they do not care about it.” (N10).*

*“I have asked to clean my hands, but the nurse is too busy or thinks it’s not important, so they do not pay much attention, and I do not feel comfortable asking again.” (N4).*

##### 3.1.3.3 Emotional burden and psychological exhaustion

Participants described experiencing emotional burden during ICU hospitalization, including anxiety, fear, sleep deprivation, and heightened stress. These emotional states were often accompanied by feelings of fatigue, distress, and psychological exhaustion. In this context, patients reported difficulty sustaining attention on self-care behaviors, including hand hygiene, and described reduced willingness or capacity to engage in additional self-care activities beyond essential treatment-related demands.

*“I’ve been really anxious and foggy-headed, and I’ve forgotten to clean my hands because I cannot focus on anything else.” (N8).*

*“This illness has hit me hard; I cannot accept being in the ICU, and I do not want to think about my hygiene issues.” (N6).*

*“I’m too tired and overwhelmed, and whenever I feel discomfort, I do not want to do anything, let alone wash my hands.” (N13).*

##### 3.1.3.4 Lack of self-efficacy and ambiguity in role perception

Participants described uncertainty regarding their role in infection prevention during ICU hospitalization. Some patients perceived hand hygiene as primarily the responsibility of healthcare providers, while others reported low confidence in performing hand hygiene independently due to physical limitations or uncertainty about correct procedures. As a result, patients expressed concerns about doing it incorrectly or interfering with medical equipment and described a preference to wait for healthcare providers rather than initiate hand hygiene themselves.

*“The nurses do most of the work for me, so I feel like whether I do it or not does not really matter.” (N3).*

*“I’m afraid I will not do it properly and might accidentally disconnect the tubes, so I’d rather wait for the nurse. “(N1).*

*“Preventing infections is something the doctors and nurses should worry about.” (N14).*

### 3.2 Improvement expectations for hand hygiene practices

#### 3.2.1 Capability-related expectations

##### 3.2.1.1 Clear and safe boundaries for initiating behaviors

Participants expressed a strong need for clear, individualized guidance on hand hygiene tailored to their medical conditions, treatment-related equipment, and physical limitations. When guidance regarding when, how, and to what extent to perform hand hygiene was unclear, patients reported hesitancy in initiating the behavior. In contrast, specific and contextualized guidance was associated with greater willingness to initiate hand hygiene and an increased sense of control over self-care behaviors.

*“I really need the nurses to tell me which situations require me to perform hand hygiene.” (N5).*

*“What I need is not just for the nurses to tell me what to do, but how to do it based on my specific condition. For example, when I’m on dialysis, what position should I maintain, and what movements are safe?” (N11).*

##### 3.2.1.2 Health education tailored to current abilities

Rather than receiving generic or information-dense education, participants described a clear preference for hand hygiene education that matched their current cognitive and physical capacities. Patients emphasized the importance of simple, accessible formats that were tailored to their individual conditions. Visual and step-by-step approaches—such as videos, images, or bedside demonstrations—were perceived as particularly helpful in facilitating understanding and building confidence in performing hand hygiene. Participants also highlighted the need to pace educational content appropriately to avoid cognitive overload.

*“It would help if there were videos or pictures clearly showing how to clean my hands, especially considering my health condition. I would feel more confident.” (N15).*

*“Please explain health knowledge in simpler terms, I cannot understand medical jargon well.” (N7).*

*“Do not overwhelm me with too much information at once. It’s hard for me to accept.” (N1).*

#### 3.2.2 Opportunity-related expectations

##### 3.2.2.1 Sustainable and easy-to-trigger behavioral prompts

Participants described relying on external cues to initiate hand hygiene during ICU hospitalization. In the context of illness-related cognitive load, medication effects, and the complex ICU environment, patients reported difficulty remembering to perform hand hygiene without timely prompts. Visual, repetitive, and non-judgmental cues—such as bedside reminder signs, short videos, or audio messages—were perceived as acceptable and effective in prompting hand hygiene initiation while minimizing discomfort or embarrassment.

*“If there were a small sign at the head of the bed, I would know when to wash my hands.” (N7).*

*“It would be helpful to have audio reminders played repeatedly to remind me of hand hygiene.” (N8).*

*“I do not want to be constantly reminded by doctors and nurses. It makes me feel embarrassed. It would be better if there were non-human reminders.” (N9).*

##### 3.2.2.2 Improving accessibility through environmental and procedural optimization

Participants described practical difficulties in accessing hand hygiene resources during ICU hospitalization and emphasized the importance of environmental and procedural arrangements that accommodate their functional limitations. Beyond the mere presence of supplies, patients stressed that hand hygiene resources needed to be consistently positioned within reach and embedded into bedside routines to support independent use. Environmental features such as designated bedside spaces for daily necessities and easy-to-use or automated hand hygiene devices were perceived as facilitating timely and feasible hand hygiene.

*“Healthcare workers should always check if patients have access to daily living supplies.” (N10).*

*“It would be helpful if there was a designated space at the bedside for our daily necessities, so we could easily reach them.” (N3).*

*“It would be great if there were automatic hand sanitizer dispensers or easy-to-access disinfectant wipes.” (N1).*

#### 3.2.3 Motivation-related expectations

##### 3.2.3.1 Enhancing motivation through role clarification and positive feedback

Participants expressed a desire for healthcare workers to clarify patients’ roles in infection prevention and emphasize the relevance of hand hygiene to their recovery. They perceived explanations, positive feedback, role modeling, and greater emphasis from healthcare providers as supportive cues that could enhance their confidence, sense of participation, and willingness to practice hand hygiene during ICU hospitalization.

*“It would help if the nurses could explain the importance of hand hygiene and infection prevention. I believe this would make patients more motivated to practice hand hygiene.” (N6).*

*“If I could participate in my treatment or self-care, instead of just lying passively, I would be more motivated to follow through on the requirements.” (N14).*

*“After I complete hand hygiene, I hope healthcare workers will praise me and provide positive feedback.” (N13).*

*“Role models are important. You could tell me about other patients who have excellent hand hygiene practices.” (N1).*

*“I hope healthcare workers take hand hygiene more seriously and help us more. I believe this will influence me to practice hand hygiene more consistently.” (N15).*

##### 3.2.3.2 Respectful communication and care practices

Participants emphasized the importance of respectful communication when being reminded to perform hand hygiene. Commanding or corrective reminders were commonly perceived as uncomfortable and discouraging, whereas respectful, empathetic, and patient-centered interactions were associated with greater willingness to engage in hand hygiene. This preference was particularly evident when patients required assistance under conditions of physical dependency.

*“When reminding me to clean my hands, please avoid using a commanding tone because it makes me feel uncomfortable and like my hygiene habits are bad.” (N10).*

*“Even in the ICU, I hope I can still have my dignity. It would be great if healthcare workers paid more attention to my hygiene situation.” (N4).*

*“When I ask for help with cleaning, I hope the nurses can be a bit more patient, otherwise, I hesitate to ask.” (N2).*

## 4 Discussion

### 4.1 The underrecognized role of awake ICU patients in infection prevention

The present study suggests that awake ICU patients may represent an underrecognized yet important component of infection prevention efforts. Although most participants acknowledged the general importance of hand hygiene, many perceived it as a secondary concern or primarily the responsibility of healthcare providers, which contributed to limited motivation for independent engagement. This perception may partially explain the low levels of patient hand hygiene observed in clinical practice. Previous evidence indicates that a substantial proportion of hospitalized patients have pathogen-contaminated hands within 48 h of admission, with some carrying multidrug-resistant organisms that may contribute to HAIs (17). Contaminated patient hands may facilitate both self-inoculation and environmental transmission through contact with medical devices, healthcare personnel, or surrounding surface (9, 18). Despite this potential role, infection prevention strategies have traditionally focused on healthcare workers, with comparatively limited emphasis on patients as active participants. An interventional study demonstrated that when patient hand hygiene compliance increased from 48 to 66%, hospital infection rates decreased from 16.9 to 9.9% (19).

However, prior research has also shown that many hospitalized patients have limited understanding of appropriate hand hygiene practices and often perform hand hygiene only when visibly dirty (10). Consistent with these findings, participants in the present study generally viewed hand hygiene as a non-priority during ICU hospitalization. Some also perceived limited emphasis on patient-performed hygiene from healthcare providers, which may further reduce willingness to engage in infection prevention behaviors. Taken together, these findings highlight that awake ICU patients should not be viewed merely as passive recipients of infection prevention measures. Instead, some patients expressed clear support needs and expectations for improving hand hygiene, suggesting that with appropriate support and encouragement, awake ICU patients may play a more active role in infection prevention during hospitalization.

### 4.2 Core characteristics of hand hygiene barriers among awake ICU patients: difficulties in behavior initiation and maintenance

The present study suggests that barriers to hand hygiene among awake ICU patients are characterized not merely by isolated obstacles but by compounded difficulties in both initiating and sustaining behavior. This pattern aligns with prior research indicating that critically ill patients face substantial self-care challenges due to physical debility, cognitive fluctuation, and environmental constraints inherent to ICU settings (20, 21).

From a capability perspective, many awake ICU patients are in the acute or early recovery phase of illness and experience physical limitations such as upper-limb weakness, pain, and restricted positioning (22, 23). In addition, fluctuations in cognitive function—often related to medication effects, sleep disruption, or environmental stress—impair patients’ attention and information processing (24), making it difficult for them to recognize appropriate moments for hand hygiene or to complete the required steps accurately.

From an opportunity perspective, structural characteristics of the ICU environment—including dense medical equipment, limited bedside space, and variable accessibility of hygiene supplies—narrow the practical window for behavior enactment. Previous research has shown that environmental accessibility is a critical determinant of patient hand hygiene adherence, and that bedside availability of alcohol-based hand rub significantly improves practice compared with education alone (25). In high-acuity settings, limited staffing and restricted family presence may further compress opportunities for timely reminders or assistance.

From a motivation perspective, although participants generally acknowledged the importance of hand hygiene, perceived infection risk often remained abstract when compared with immediate survival concerns. Similar observations have been reported in critical care contexts, where preventive behaviors are deprioritized under conditions of psychological distress and treatment intensity (26). Emotional burden and diminished self-efficacy may therefore weaken behavioral persistence even when awareness exists (27, 28).

Importantly, capability-, opportunity-, and motivation-related factors often coexisted and may interact, rather than operate independently. Physical limitations could increase reliance on external support, limited opportunities may reduce confidence, and low confidence may further challenge motivation. Together, these factors may contribute to a heightened behavioral burden, making hand hygiene a cognitively and physically demanding task for awake ICU patients. This burdened context may help explain persistently low adherence despite patients’ general awareness of the importance of hand hygiene.

### 4.3 Recommendations for improving hand hygiene practices among awake ICU patients

Despite the substantial barriers identified in this study, participants commonly expressed a desire for clearer guidance, practical support, and respectful communication to facilitate hand hygiene during ICU hospitalization. These expectations suggest that awake ICU patients are not merely passive recipients of care, but may be more willing to participate in hand hygiene practices when supportive conditions are provided.

Participants emphasized the importance of situation-specific guidance tailored to their physical and cognitive capacities, and noted that simplified and individualized education could help them better understand and follow hand hygiene practices. Traditional health education may overwhelm patients with excessive information, reducing their receptiveness to hand hygiene education (29). In ICU settings, where patients’ capabilities and cognitive functions may be limited, health education may therefore benefit from moving beyond general knowledge delivery to provide clear and contextualized guidance adapted to patients’ physical and cognitive conditions. Participants also described a need for learning about appropriate hand hygiene methods and key moments for action. Research has shown that alcohol-based hand sanitizers or disinfectant wipes can provide effective hand hygiene when traditional washing is not feasible (30). Clear guidance regarding the use of these alternatives, as well as key moments such as before meals, after toileting, or after touching medical devices, may help reduce uncertainty and support adherence (31, 32). Personalized bedside instructions and demonstrations adapted to patients’ positioning and functional abilities may further help make hand hygiene guidance practical, understandable, and relevant. These observations suggest that awake ICU patients may be more willing to participate in hand hygiene practices when guidance is responsive to their individual needs.

Structural constraints within the ICUs in China, including limited family presence and persistent workforce shortages, substantially limit patients’ opportunities to perform hand hygiene. Participants described that these contextual realities often reduced opportunities for continuous assistance or timely reminders. In this environment, ensuring that hand hygiene resources, such as alcohol-based sanitizers or disinfectant wipes, are readily accessible within patients’ immediate reach may help support engagement and adherence (11). In addition, some participants noted that non–human-dependent and sustained behavioral prompts may be particularly valuable in settings with limited staffing and restricted visitation. Fixed visual cues (e.g., bedside signage) and simple electronic reminders can provide timely triggers for action without increasing the workload of healthcare providers or relying on family involvement (33).

Hand hygiene practices among awake ICU patients were closely related to their understanding of the importance of hand hygiene and their willingness to participate. Some participants perceived hand hygiene primarily as the responsibility of healthcare providers rather than as a personal protective behavior. In addition, some participants perceived limited attention to patient-performed hand hygiene from healthcare providers, which may further reduce patients’ willingness to participate independently. These narratives highlight that patients’ willingness to participate in hand hygiene may be influenced not only by their own awareness, but also by the extent to which healthcare providers emphasize patient-performed hand hygiene. Previous research has shown that training and education can enhance healthcare providers’ awareness of the importance of hand hygiene, enabling them to consistently communicate its relevance to patients’ health outcomes and infection prevention (34), which may help patients better recognize the importance of hand hygiene and increase their willingness to participate. Patients’ self-efficacy plays a key role in hand hygiene behavior. Healthcare providers can foster self-efficacy by offering empathetic communication, practical demonstrations, achievable goals, and positive feedback (35). Emotional burden and environmental discomfort were also described as important influences on participation. Anxiety, sleep disruption, noise, and environmental stress may further reduce patients’ attention and motivation toward self-care behaviors, including hand hygiene. In this context, supportive ICU environments that reduce unnecessary stressors and improve patient comfort may help facilitate participation in hand hygiene practices (36). Overall, supporting patient participation in hand hygiene may require not only improving patients’ understanding of hand hygiene, but also creating supportive conditions that strengthen motivation, confidence, and engagement during ICU hospitalization.

### 4.4 Strengths and limitations

The study provides novel qualitative insights into hand hygiene practices among awake ICU patients, a population that has received limited attention in infection prevention research. By applying the COM-B model, the study offers a theoretically grounded understanding of the capability-, opportunity-, and motivation-related challenges and support needs shaping patient hand hygiene in ICU settings. By focusing on patients’ lived experiences, the study identifies both barriers and improvement expectations, contributing to a deeper understanding of patient participation in hand hygiene during ICU hospitalization.

Several limitations should be acknowledged. First, participants were recruited from selected ICU units within a single tertiary hospital in China. Therefore, the findings should be interpreted within the specific organizational and clinical context of this setting. Second, cognitive fluctuations and stress associated with critical illness in ICU settings may have influenced the accuracy of reported hand hygiene behaviors. Third, the exclusion of healthcare providers’ perspectives may have limited a more relational understanding of patient–provider dynamics in hand hygiene practices.

Future research should incorporate multi-center designs and combine qualitative insights with observational or behavioral measures of patient hand hygiene participation. Including healthcare providers’ perspectives may also support the development of more integrated and context-sensitive approaches to patient hand hygiene in ICU settings.

## 5 Conclusion

This study suggests that hand hygiene among awake ICU patients should not be understood solely as an issue of patient knowledge or behavioral compliance. Within the ICU context, patients’ ability to engage in hand hygiene is influenced by illness-related functional limitations, environmental support, and emotional and motivational challenges. Importantly, patients also expressed support needs and a willingness to participate in infection prevention. These findings suggest that improving patient hand hygiene in ICU settings may require not only responsive support from healthcare providers, but also active efforts to facilitate and promote patient participation.

## Data Availability

The datasets presented in this article are not readily available due to the qualitative nature of this study and the need to protect participant privacy and confidentiality. Requests to access the datasets should be directed to ccutaolin@163.com.
