# Supplementary material for: Barriers to hand hygiene practices and improvement expectations among awake ICU patients: a qualitative study
Source: Front Public Health. 2026 Jun 2;14:1814268. doi: 10.3389/fpubh.2026.1814268 (PMC13269352; doi:10.3389/fpubh.2026.1814268)
Supplement: Supplementary file 1 [file Table_1.DOCX]

Supplementary File 1

Consolidated Criteria for Reporting Qualitative Research (COREQ): 32-item Checklist

| **No. Item** | **Guide questions/description** | **Answer** | **Reported in manuscript** |
| --- | --- | --- | --- |
| Domain 1: Research team and reﬂexivity |  |  |  |
| Personal Characteristics |  |  |  |
| 1.Interviewer*/*facilitator | Which author*/*s conducted the interview or focus group? | YY | 2.4 Data collection |
| 2. Credentials | What were the researcher’s credentials? | ICU nurse with qualitative research training | 2.7 Rigor and reflexivity |
| 3. Occupation | What was their occupation at the time of the study? | ICU nurse | 2.7 Rigor and reflexivity |
| 4. Gender | Was the researcher male or female? | Female | 2.4 Data collection |
| 5.Experience and training | What experience or training did the researcher have? | All researchers have received training in qualitative research methods | 2.4 Data collection |
| Relationship with participants |  |  |  |
| 6. Relationship established | Was a relationship established prior to study commencement? | No prior relationship with participants | 2.4 Data collection |
| 7. Participant knowledge of the interviewer | What did the participants know about the researcher? e.g. personal goals, reasons for doing the research | Participants were informed of the study purpose, procedures, and confidentiality before interviews | 2.4 Data collection |
| 8.Interviewer characteristics | What characteristics were reported about the interviewer/facilitator? e.g. Bias, assumptions, reasons and interests in the research topic | Reflexive discussions were conducted throughout data collection and analysis to minimize prior assumptions | 2.7 Rigor and reflexivity |
| Domain 2: study design |  |  |  |
| Theoretical framework |  |  |  |
| 9.Methodological orientation and Theory | What methodological orientation was stated to underpin the study? e.g. grounded theory, discourse analysis, ethnography, phenomenology, content analysis | Qualitative descriptive design, with deductive content analysis informed by the COM-B model | 2.1 Design;  2.5 Data analysis |
| Participant selection |  |  |  |
| 10.Sampling | How were participants selected? e.g. purposive, convenience, consecutive, snowball | Purposive sampling | 2.2 Setting and participants |
| 11.Method of approach | How were participants approached? e.g. face-to-face, telephone, mail, email | Face-to-face | 2.2 Setting and participants |
| 12.Sample size | How many participants were in the study? | 15 | 3 Results |
| 13. Non-participation | How many people refused to participate or dropped out? Reasons? | Two eligible patients declined participation because of fatigue | 2.2 Setting and participants |
| Setting |  |  |  |
| 14.Setting of data collection | Where was the data collected? e.g. home, clinic, workplace | Bedside in adult ICU units | 2.4 Data collection |
| 15. Presence of non-participants | Was anyone else present besides the participants and researchers? | No | 2.4 Data collection |
| 16.Description of sample | What are the important characteristics of the sample? *e.g. demographic data, date* | Participant characteristics presented in Table 1 | Table 1 |
| Data collection |  |  |  |
| 17.Interview guide | Were questions, prompts, guides provided by the authors? Was it pilot tested? | Yes. Pilot tested in two patients | 2.3 Interview guide |
| 18. Repeat interviews | Were repeat interviews carried out? If yes, how many? | No | 2.4 Data collection |
| 19.Audio/visual recording | Did the research use audio or visual recording to collect the data? | Yes. Audio recording | 2.4 Data collection |
| 20.Field notes | Were ﬁeld notes made during and/or after the interview or focus group? | Yes | 2.4 Data collection |
| 21. Duration | What was the duration of the interviews or focus group? | Approximately 30–40 minutes | 2.4 Data collection |
| 22. Data saturation | Was data saturation discussed? | Yes | 2.2 Setting and participants; 2.5 Data analysis |
| 23. Transcripts returned | Were transcripts returned to participants for comment and/or correction? | No | 2.7 Rigor and reflexivity |
| Domain 3: Analysis and findings |  |  |  |
| Data analysis |  |  |  |
| 24. Number of data coders | How many data coders coded the data? | 2 coders | 2.5 Data analysis |
| 25.Description of the coding tree | Did authors provide a description of the coding tree? | No | N/A |
| 26. Derivation of themes | Were themes identiﬁed in advance or derived from the data? | Themes were informed by the COM-B framework and refined through iterative analysis | 2.5 Data analysis |
| 27. Software | What software, if applicable, was used to manage the data? | NVivo 12 | 2.5 Data analysis |
| 28.Participant checking | Did participants provide feedback on the ﬁndings? | No | 2.7 Rigor and reflexivity |
| Reporting |  |  |  |
| 29. Quotations presented | Were participant quotations presented to illustrate the themes */* ﬁndings? Was each quotation identidied? E.g. participant number | Yes. Participant identifiers provided | 3 Results |
| 30. Data and findings consistent | Was there consistency between the data presented and the ﬁndings? | Yes | 3 Results |
| 31.Clarity of major themes | Were major themes clearly presented in the ﬁndings? | Yes | 3 Results |
| 32.Clarity of minor themes | Is there a description of diverse cases or discussion of minor themes? | Yes | 3 Results  4 Discussion |

N/A: not applicable.

Developed from:

Tong A, Sainsbury P, Craig J. Consolidated criteria for reporting qualitative research (COREQ): a 32-item checklist for interviews and focus groups. *Int J Qual Health Care* (2007) 19:349–357. doi: 10.1093/INTQHC/MZM042
